# Processes for the 3D Printing of Hydrodynamic Flow-Focusing Devices

**DOI:** 10.3390/mi14071388

**Published:** 2023-07-07

**Authors:** Diwakar M. Awate, Seth Holton, Katherine Meyer, Jaime J. Juárez

**Affiliations:** 1 Department of Mechanical Engineering, Iowa State University, Ames, IA 50011, USA; 2 Center for Multiphase Flow Research and Education, Iowa State University, Ames, IA 50011, USA

**Keywords:** 3D printing, flow focusing, millifluidics

## Abstract

Flow focusing is an important hydrodynamic technique for cytometric analysis, enabling the rapid study of cellular samples to identify a variety of biological processes. To date, the majority of flow-focusing devices are fabricated using conventional photolithography or flame processing of glass capillaries. This article presents a suite of low-cost, millifluidic, flow-focusing devices that were fabricated using a desktop sterolithgraphy (SLA) 3D printer. The suite of SLA printing strategies consists of a monolithic SLA method and a hybrid molding process. In the monolithic SLA approach, 1.3 mm square millifluidic channels were printed as a single piece. The printed device does not require any post processing, such as bonding or surface polishing for optical access. The hybrid molding approach consists of printing a mold using the SLA 3D printer. The mold is treated to an extended UV exposure and oven baked before using PDMS as the molding material for the channel. To demonstrate the viability of these channels, we performed a series of experiments using several flow-rate ratios to show the range of focusing widths that can be achieved in these devices. The experiments are validated using a numerical model developed in ANSYS.

## 1. Introduction

Hydrodynamic focusing is a process whereby a sample entrained by a fluid core is introduced to a channel bound by a secondary fluid, known as a fluid sheath. Adjusting the relative flow rates of the core and the sheath minimizes mixing between the two elements, while controlling the width of the core [1]. Hydrodynamic flow focusing plays an important role in controlling the passage of chemical and biological samples through microfluidic devices for a variety of applications, such as cell/particle counting and sorting in flow cytometers [2,3,4,5,6,7,8,9,10], diffusion-based mixers [11,12,13], cell patterning [14], receptor-ligand assays and DNA-hybridization [15], DNA stretching [16], micro flow switches [17,18,19,20], and bubble or droplet generators [21,22]. 

A wide variety of techniques are available for fabricating flow-focusing devices, such as replica molding on a silicon wafer master [23,24] (i.e., soft lithography), polymer injection molding [25], hot embossing of a thermoplastic [26], wet or dry etching of silicon or glass substrates [27], and micromachining polymer substrates [28]. The disadvantage of many of these techniques lies in the need to access capital equipment and facilities, which are often prohibitively expensive [29]. Furthermore, mastering these techniques can be a complex and time-consuming process, requiring specialized training in microfabrication [30]. 

Alternative methods for fabricating flow-focusing devices have been examined to reduce the complexity of fabrication. One process utilizes the application of a flame to the tip of a clean-cut millimetric glass capillary to create a nozzle for flow focusing [31]. While the process is simple compared to the fabrication processes described above, this method lacks precise control over nozzle dimensions. Acoustic standing waves have also been used to focus samples for analysis [32,33,34]. In this approach, acoustic waves utilize acoustophoresis for focusing samples. Acoustic fields work well for samples consisting of particles of ~1 μm. Below this size limit, secondary acoustic effects, such as acoustic streaming, influence the motion of particles and limit the ability to control focusing [35].

Thus, it is critical to identify a simple process for fabricating flow-focusing devices that addresses many of the issues described above. One such method is to utilize 3D printing (3DP) as a technique for creating flow-focusing devices. Three-dimension printing is a mature fabrication approach that is being used to create a variety of microfluidic devices [36,37,38,39]. This fabrication technique has the advantage of being highly cost-effective [40,41], while the implementation of 3DP may serve as a platform for chemical and biological discovery in a variety of laboratory environments [42,43,44]. Three-dimensional printing and associated additive manufacturing techniques (e.g., sterolithography) now form the basis for STEM curricula in K-12 and university environments [45,46,47], thus making it easier to train the current generation of scientists and engineers in its application to microfluidic device fabrication.

This paper aims to characterize stereolithography (SLA) 3D printing as a method for fabricating low-cost flow-focusing devices. We devised two separate SLA fabrication strategies to assess the ability of a low-cost desktop SLA printer to fabricate flow-focusing devices. The first approach utilizes the monolithic SLA method, whereby 1.3 mm square channels were printed as a single piece in 15 min using a desktop SLA printer. The printed device does not require any post-processing such as bonding or surface polishing for optical access. The second process utilizes a hybrid molding approach to create the flow-focusing device. In this approach, the mold is 3D printed using SLA followed by a curing procedure that relies on extended UV exposure and substrate baking in a laboratory oven. Once the process is complete, PDMS is poured over the mold and allowed to cure, forming a device with impressions of a channel that could be bonded to glass. Numerical simulations were performed to test the hydrodynamic focusing capabilities of the devices, followed by lab experiments at different sheath flow rates to assess device performance. The experiments performed a different sheath flow rates show that our devices are capable of focusing fluid cores to widths that range from 60 mm up to 500 mm. The numerical simulations show excellent agreement with the experiments. Overall, this paper demonstrates two unique SLA 3D printing-based, cost-effective fabrication approaches for microfluidic devices designed for hydrodynamic focusing. 

## 2. Materials and Methods

### 2.1. Monolithic SLA Method

**Figure 1:**
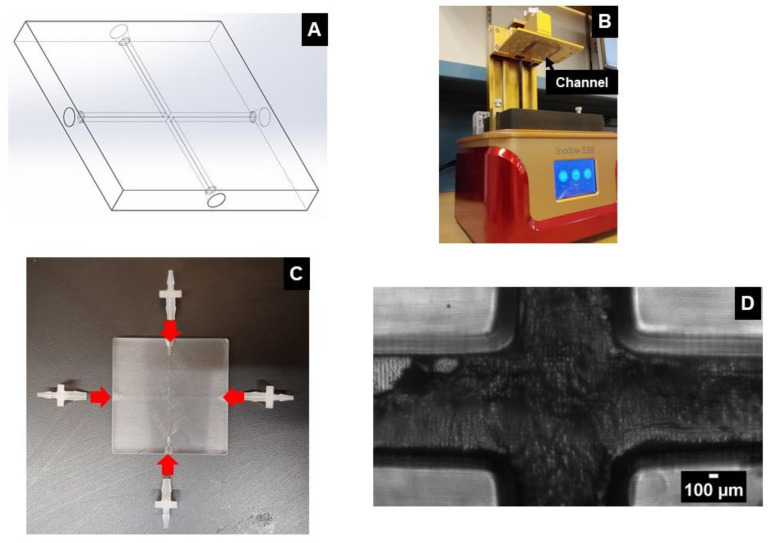
(**A**) 3D CAD model of the milli-channel. (**B**) SLA printer used for printing the channel. (**C**) Image showing the connectors that need to be glued to the channel to attach tubing for experimental use (**D**) Microscopic image of the channel junction.

The first method explored by this paper utilizes stereolithography (SLA) as a fabrication method for printing entire channels as a single piece (i.e., as a single block or monolith); hence, we refer to this approach as the monolithic SLA method. This 3D-printed channel was designed using Solidworks (Dassault Systemes S.A.), as illustrated in Figure 1A, and then printed with a low-cost (~$179) 3D printer Shadow 5.5S (QIDI Technology Ltd., Ruian, China) (Figure 1B). The SLA UV-curing resin (part number POT061, Anycubic, Shenzhen, China) costs $35/kg, which makes it affordable and reduces the overall cost of channel fabrication. SLA resins are available in clear form which makes the printed channels transparent and allows researchers to observe the fluid flow inside the channel using a microscope. 

The device produced by our monolithic SLA approach features a device that is 50 mm by 50 mm and is 6 mm thick. Four channels, 1.3 mm square, are left as cavities inside the device and meet in the center. The channel inlet ports feature a conical structure at the end to accommodate a commercial 1/16 inch barbed fitting in order to hold a commercial tubing (1/16 inch OD and 0.007 inch ID), through which the sample and sheath flows are introduced. This CAD model was converted into .ctb file using CHITUBOX software (CBD-Tech, Shenzhen, China) for SLA printing. After printing the device, it was left submerged into IPA for several hours to clean excess resin from channel cavities. Once the device was clean, the barbed fittings were glued to the channel openings using epoxy as shown in Figure 1C. The device was checked for any leaks by flowing water through channel cavities. Finally, the device was examined under a microscope to check possible defects as shown in Figure 1D.

### 2.2. Hybrid Molding Method

**Figure 2:**
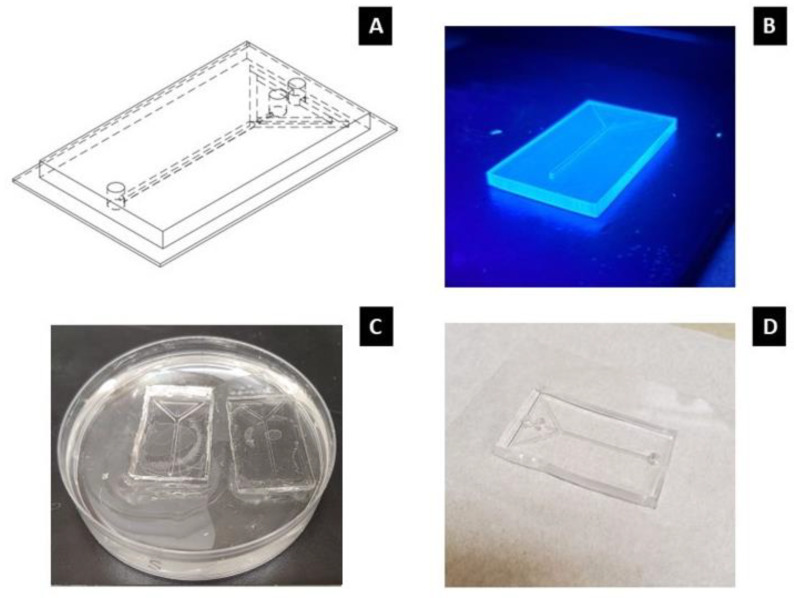
(**A**) 3D CAD model of the microchannel. (**B**) Device mold placed under UV light. (**C**) Microchannels placed in a Petri dish filled with PDMS. (**D**) Final version of microchannel.

The second method we devised for creating channels relies on a hybrid fabrication approach, whereby an impression of the channel is printed as a mold. The Solidworks design of the channel in Figure 2A differs from the one presented in Figure 1A, as the aim of the process was to ultimately bond an impression of the mold to a 2 in by 3 in glass slide (Fisher Brand, catalog no. 12-550C). Thus, this method is constrained by the substrate it will be bonded to. After printing, the channel was then cleaned with ethanol and placed under a UV light for 1 h (Figure 2B). Halfway through the UV process, the mold was flipped over to expose the bottom of the mold to UV light. Following the UV light process, the model was baked for at least 2 h in an oven at 120 °C before being baked for an additional 12 h at 60 °C. These additional curing and baking steps were implemented because it was discovered that the molding material, polydimethylsiloxane (PDMS), would not properly crosslink when in contact with the mold without these steps.

After the curing process, the mold was placed in a Petri dish with the channel impressions facing up (Figure 2C). PDMS (Sylgard 184, Dow Chemical) was mixed in a 10:1 ratio and poured over the resin print, filling the Petri dish about half-way. Bubbles that developed during the mixing process were removed by placing the Petri dish in a vacuum chamber for at least 30 min. After being stored at 25 °C for at least 24 h, the PDMS cured directly on the mold, from which the channel could be extracted. Inlet/outlet holes were created in the device using a biopsy punch. To prepare the device for plasma bonding, scotch tape and compressed air were used to clean the underside of the device and a glass slide. Both were placed in the plasma cleaner with the bonding sides face-up. A vacuum was formed in the chamber and oxygen gas was introduced. The device and glass slide were exposed to plasma for roughly 40 s before the process was complete. The device was then pressed by hand onto the glass slide, forming a permanent bond between the surfaces. A 3 cm long, 2.5 mm-diameter silicone tubing was placed into each inlet/outlet hole and glued to the device with additional PDMS (Figure 2D).

### 2.3. Sample Preparation

For both millifluidic channel devices, opaque samples of water mixed with fluorescent dye rhodamine B (R6626, Sigma-Aldrich, Milwaukee, WI, USA) were used to be able to distinguish the sample flow from sheath flow on microscope imaging. The dye sample was prepared by adding 0.2 g of rhodamine B to 10 mL of deionized (DI) water, sourced from an ARIES High-Purity Water System with a 0.2 μm filter (Aries Filterworks, Camden, NJ, USA). The sample was prepared and kept in a 50 mL centrifuge tube until further use. 

### 2.4. Experimental Setup

**Figure 3:**
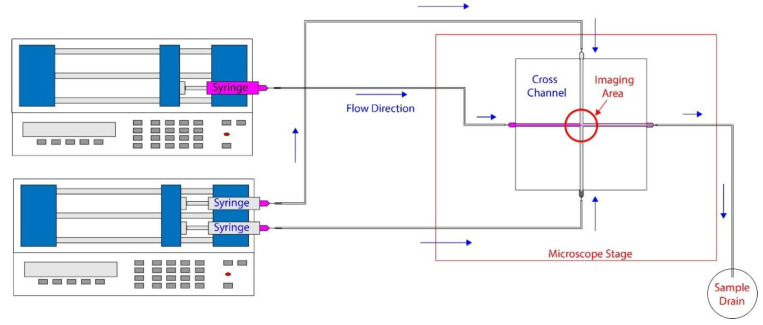
A schematic representation of the set-up used in our experiments.

The experimental setup is schematically shown in Figure 3. The dyed sample was placed on a vortex mixer for 45 s prior to the start of each experiment. Then, it was drawn into a 10 mL syringe (Becton-Dickinson, Franklin Lakes, NJ, USA) and placed on a syringe pump (Advance 1200, CellPoint Scientific, Gaithersburg, MD, USA). The sheath flow was provided using two 10 mL syringe filled with deionized (DI) water and pumped using another syringe pump (Fusion 100, Chemyx, Stafford, TX, USA). The microfluidic device was then connected to syringes with tubing, as shown in the schematics and then placed on a microscope (Olympus IX70) stage. The microscopic images of hydrodynamic focusing were captured using a 4× microscope objective. 

## 3. Modeling and Simulation

A two-fluid model, first developed by Yang and Hsieh [48], was utilized to simulate the flow transport behavior of the sample and sheath streams inside a hydrodynamic flow-focusing device like the ones fabricated above. We provide a brief overview of the model here. In this analysis, both sample and sheath fluids are treated as two-dimensional, laminar, incompressible, and isothermal flows over the computational domain. The model consists of two sets of transient conservation equations of mass and momentum with the consideration of the interfacial momentum exchange between two fluids. The macroscopic flow properties can be determined by determining the amount of sample (subscript s below) and sheath (subscript sh below) relative to the total amount entering the device (i.e., volume fraction). To describe the focusing-flow field, the present analysis deals with the full Navier–Stokes model for resolving high-shear rate and recirculating flows. The governing equations were stated as follows:

Continuity equation for the sample fluid:

(1) $$\frac{𝜕\left(\alpha_{s}\rho_{s}\right)}{𝜕t}+\nabla .\left(\alpha_{s}\rho_{s}{\vec{u}}_{s}\right)=0$$

Continuity equation for the sheath fluid:

(2) $$\frac{𝜕\left(\alpha_{sh}\rho_{sh}\right)}{𝜕t}+\nabla .\left(\alpha_{sh}\rho_{sh}{\vec{u}}_{sh}\right)=0$$

Momentum equation for the sample fluid:

(3) $$\frac{𝜕\left(\alpha_{s}\rho_{s}{\vec{u}}_{s}\right)}{𝜕t}+\nabla .\left(\alpha_{s}\rho_{s}{\vec{u}}_{s}{\vec{u}}_{s}\right)=-\alpha_{s}\nabla P+\nabla .\alpha_{s}\tau_{s}+\alpha_{s}\rho_{s}\vec{g}+{\vec{M}}_{s}$$

Momentum equation for the sheath fluid:

(4) $$\frac{𝜕\left(\alpha_{sh}\rho_{sh}{\vec{u}}_{sh}\right)}{𝜕t}+\nabla .\left(\alpha_{sh}\rho_{sh}{\vec{u}}_{sh}{\vec{u}}_{sh}\right)=-\alpha_{sh}\nabla P+\nabla .\alpha_{sh}\tau_{sh}+\alpha_{sh}\rho_{sh}\vec{g}+{\vec{M}}_{sh}$$

The variables, $\rho ,\;\alpha ,\;\vec{u},\;P,\;\tau ,\;\vec{g}$, and $\vec{M}$ denote density, volume fraction, velocity vector, pressure, stress tensor, gravitational acceleration, and interfacial momentum exchange rate, respectively. The subscripts _s_ and _sh_ denote the sample and sheath fluids, respectively. Since only two kinds of fluids are present in the flow field, the sum of the volumetric fractions for sample and sheath fluids are equal to unity (i.e., $\alpha_{s}+\alpha_{sh}=1$).

**Figure 4:**
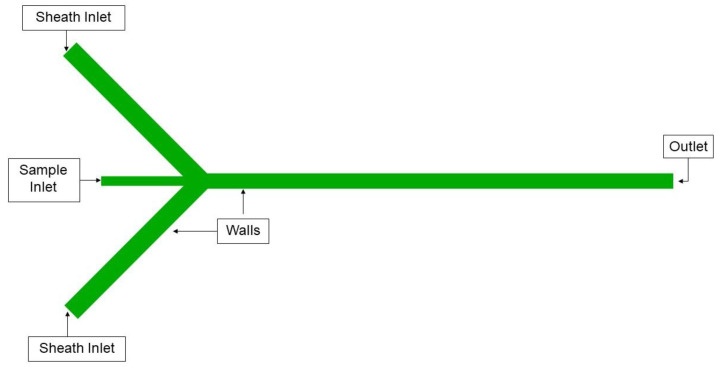
Diagram showing the hybrid channel model simulated in ANSYS Fluent.

**Figure 5:**
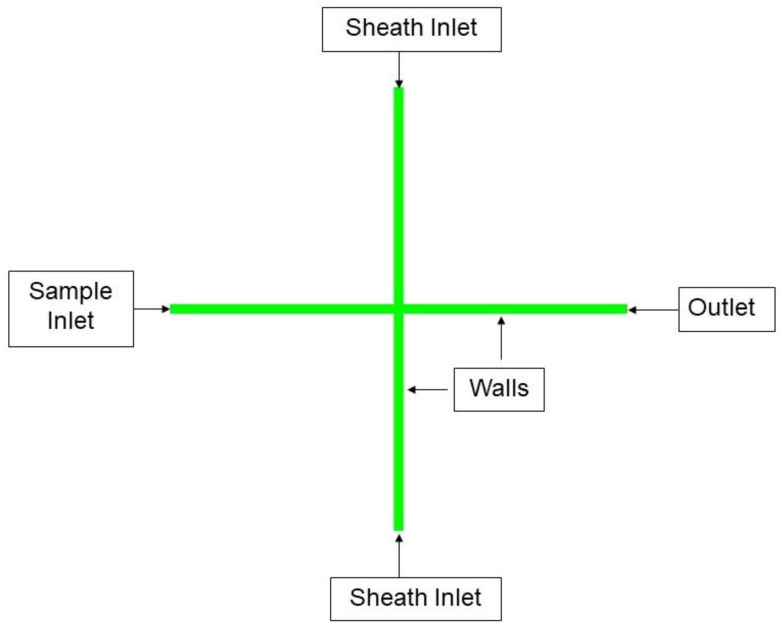
Diagram showing the monolithic channel model simulated in ANSYS Fluent.

**Figure 6:**
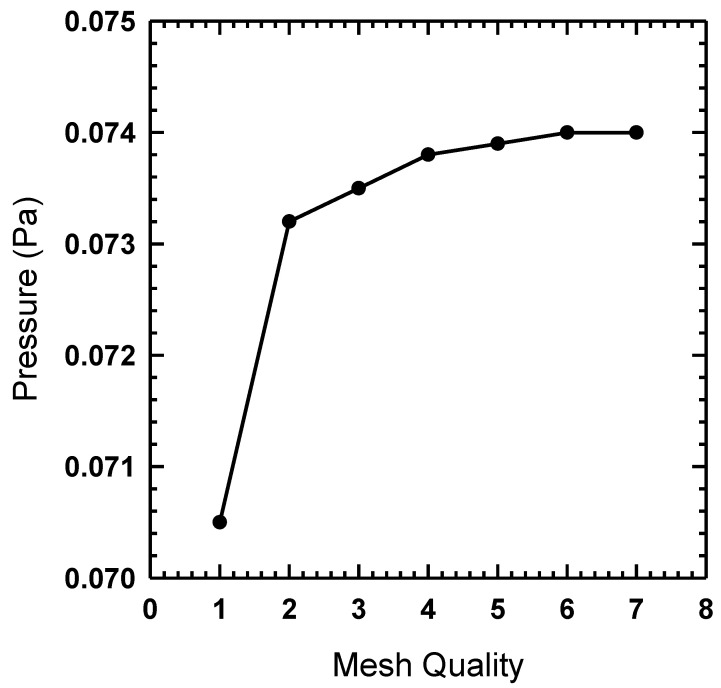
Study showing mesh independence in the ANSYS Fluent model.

A simulation utilizing Equations (1)–(4) was carried out in ANSYS Fluent with the Volume of Fluid (VOF) approach [49], which allows us to model the hydrodynamic focusing capabilities of the 3D printed channel. To perform a VOF approach, the governing partial differential equations need to be integrated over discrete cells formed by the numerical grids. The 2D geometry (Figure 4) for the monolithic channel was built using ANSYS SpaceClaim and the computational mesh was created using ANSYS meshing module, as shown in Figure 5. A mesh independence study was carried out, as shown in Figure 6. The final mesh was built using 60 µm four node quadrilateral elements, and inflation layers were added to the near-wall region to accurately capture the velocity gradient within the boundary layer. 

Boundary conditions were applied to the model using ANSYS Fluent. The locations where boundary conditions were applied are shown in Figure 5. Similarly, the model was built for the hybrid channel (Figure 4). There are two inlets for sheath flow (water) and a third one for sample flow. These inlets were specified as pressure inlets with the flow direction perpendicular to the boundary. Predetermined flow velocities were then set on these inlets for different simulation cases. The flow entering these three inlets exits through a common pressure outlet. All other edges of the model were defined as stationary walls with no slip condition. 

**Figure 7:**
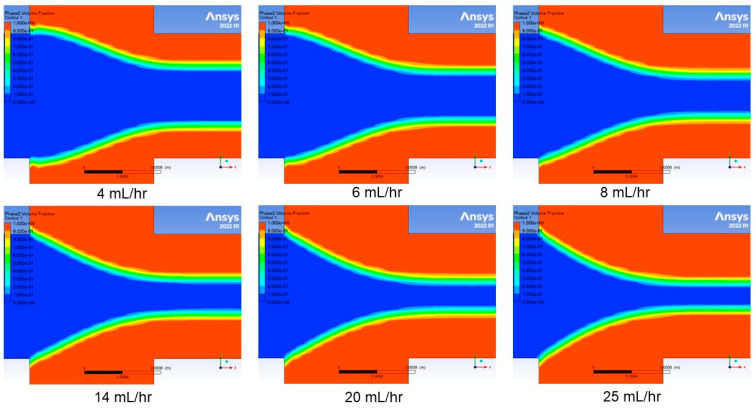
Numerical simulation results for the monolithic channel at different sheath flow rates.

**Figure 8:**
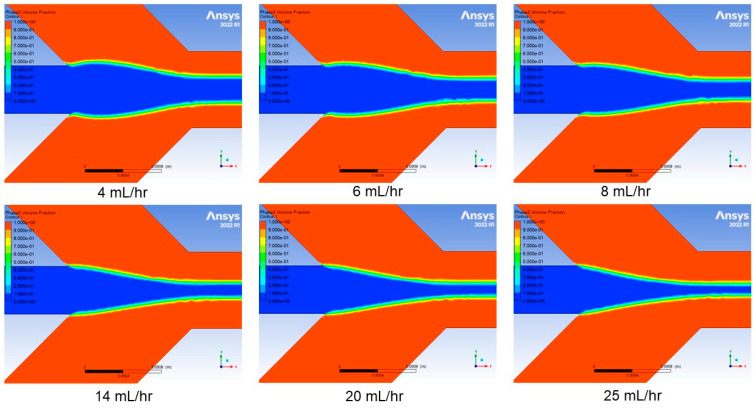
Numerical simulation results for the hybrid channel at different sheath flow rates.

A pressure based solver with relative velocity formulation was used to account for fluid motion. A time-dependent solver is used when setting up simulation with volume of fluid model, although we were only concerned with steady-state hydrodynamic focusing. A laminar model was chosen as most representative of the fluid dynamics in the microchannel. This model was combined with the VOF model to represent two co-moving fluids by solving a single set of momentum equations and tracking the volume fraction of each fluid throughout the domain. This process enables visualization of the volume fraction results at the end of the simulation (Figure 7 and Figure 8). The simulation cases were based on variations in sheath flow rate while keeping the sample flow rate constant at 1 mL/h. These six simulation cases have sheath flow rates at 4, 6, 8, 14, 20, and 25 mL/h, which are representative of the experimental results obtained in this work.

## 4. Results and Discussion

### 4.1. Effect of Sheath Flow Rate

The simulation results for the monolithic channel and hybrid channel are shown in Figure 7 and Figure 8, respectively. The simulation cases were based on variations in sheath flow rate while keeping the sample flow rate constant at 1 mL/h. There were six simulation cases set up with sheath flow rates at 4, 6, 8, 14, 20, and 25 mL/h. The simulation results predicted that with a constant sample core-flow rate, the hydrodynamic focusing width decreases as the sheath flow rate increases.

### 4.2. Experimental Results

**Figure 9:**
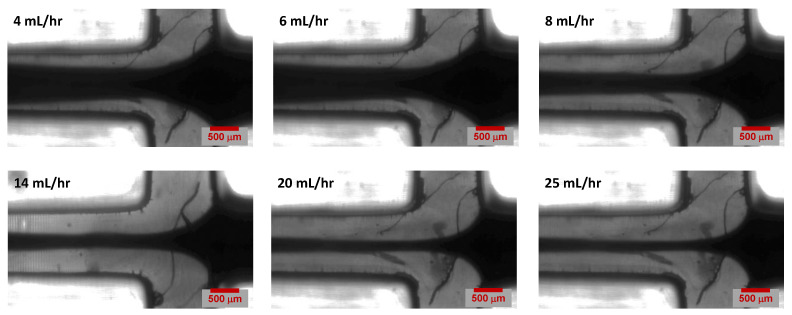
Optical microscopy images of hydrodynamic focusing obtained for various flow rate conditions in a 3D printed monolithic channel.

Experiments were performed with a fixed core-flow rate of 1 mL/h, while changes in the core focusing width were observed for six different sheath flow rates (4 mL/h, 6 mL/h, 8 mL/h, 14 mL/h, 20 mL/h and 25 mL/h). Three experiments were performed for each flow rate The microscopic images of the T-channel junction are shown in Figure 9. It can be observed in the microscopy images that the hydrodynamic-focusing width narrows with increasing sheath flow rate, as predicted by our simulation results in Section 4.1. 

The focusing width is identified using the full width at half maximum procedure, where the focusing width is taken as the region where the local intensity drops below half of the maximum intensity of the focused stream. The procedure for extracting the width begins by taking a grayscale image and inverting the color scheme so that the dark dye in the focusing stream appears as white, while all areas that are brighter than the dye appear as black or grey. We follow this up with a thresholding procedure that divides these images into two parts, background and foreground, based on a specific intensity value or range of values. This helps separate the objects of interest from the background in an image. The next step is to draw a vertical line over the image as shown in Figure S2 in Supplementary Materials. Then, an intensity profile could be obtained of the pixels along that line which creates a gaussian curve that allows us to calculate the focusing width for that particular image using the FWHM principle (see Figure S1). Finally, we combined all six microscopic images and drew a single vertical line over it to make sure that we were obtaining FWHM measurements at the same spot in all images.

**Figure 10:**
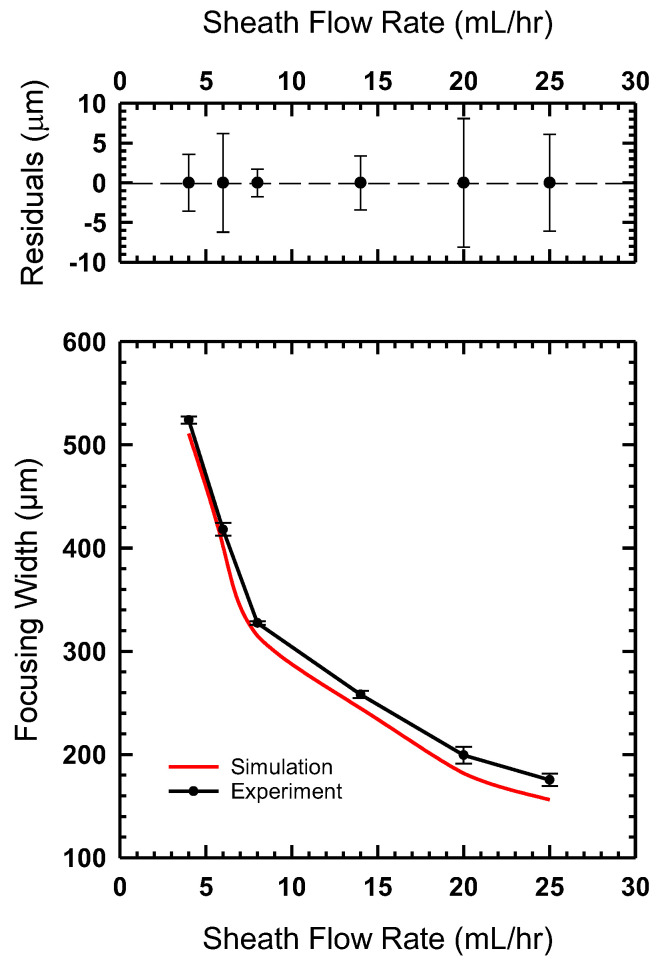
(**Bottom**) The flow focusing width measured for the device printed as a monolith compared to the simulations shown in Figure 7. (**Top**) The residual plot shows that measured focusing widths vary from ±5 mm or less for most experiments.

The process for estimating flow focusing width was also applied to our simulation data. The comparison of experimental and simulation data is shown in Figure 10. We find that the flow focusing width decreases with an increase in sheath flow rate. Figure 10 shows excellent agreement between our simulation and experimental results. We also found that the focusing width was highly repeatable with extremely narrow error bars, as shown in the residual plot in Figure 10.

**Figure 11:**
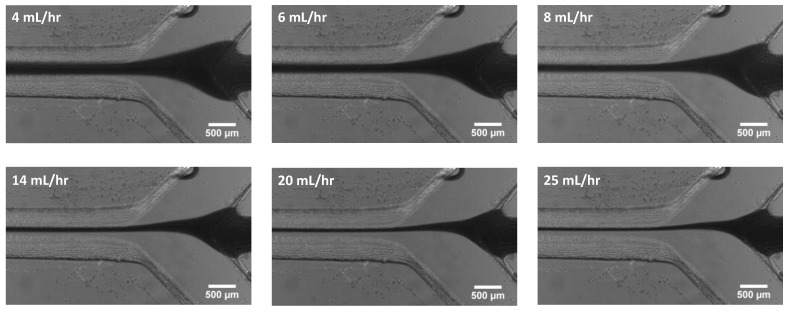
Microscopic images demonstrating the effect of varying sheath flow rate on the width of the focused sample stream.

**Figure 12:**
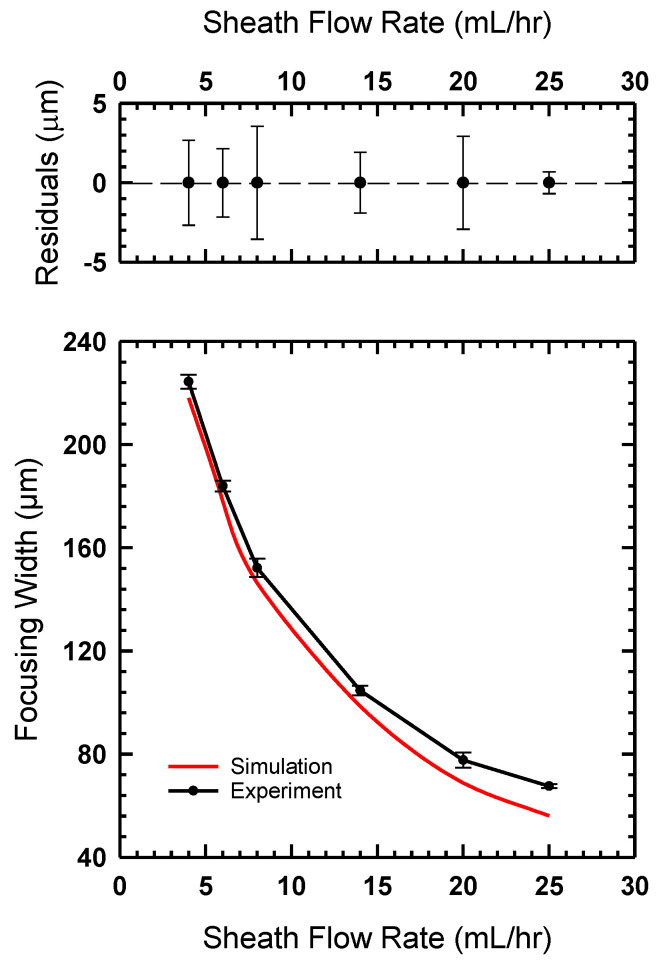
(**Bottom**) The flow-focusing width measured for the device, fabricated using the hybrid method compared to the simulations shown in Figure 8. (**Top**) The residual plot shows that measured focusing widths vary from ±4 mm or less for most experiments.

A similar set of experiments based on increasing the sheath flow rate were performed for the channel created via the hybrid method. The same flow conditions were used for these experiments. Microscope images were captured and are shown in Figure 11. The hydrodynamic focusing width was measured using the same FWHM method used for previous experimental results. The focusing width results were plotted and are shown in Figure 12. We observe a similar agreement between our experimental observations and the simulation results. In comparing this hybrid approach to the monolithic SLA approach, we find that we achieve better focusing with the hybrid approach. This could be a result of the slightly different geometries used in both experiments. As discussed in the methods section above, this design was chosen due to substrate limitations. A future study comparing the angle of incoming channels could help identify how this difference influences focusing width.

## 5. Conclusions

In this work, we presented two different hydrodynamic flow-focusing devices built using two unique fabrication approaches based on stereolithography (SLA). The monolithic SLA method involves printing the whole device as one piece, which does not need any further assembly or processing such as polishing the surface for optical access. The device was printed in 15 min and required 4.8 mL of clear SLA resin. The printed device is reusable, which makes it cost-effective for flow-focusing applications. The second device was fabricated with the hybrid molding method. First, a mold was 3D printed and cured under UV light, followed by a heating process inside an oven. It was finally encapsulated in PDMS and then bonded to a glass slide using plasma bonding. 

Numerical simulation was set up using ANSYS Fluent to check the hydrodynamic focusing capabilities of the 3D printed channel. The simulation cases were based on six different sheath flow rates (4, 6, 8, 14, 20, and 25 mL/h) while keeping the sample flow rate constant at 1 mL/h. The simulation results predicted that, with a constant sample flow rate, the hydrodynamic focusing width decreases with an increased sheath flow rate. Samples were prepared using dyed DI water to distinguish sheath flow from sample flow and lab experiments were performed at the same sheath flow rates as per the numerical simulation. Microscopic images of the channel junction were taken and processed using FWHM principle to calculate the hydrodynamic-focusing width at each sheath flow rate. It was observed that the hydrodynamic-focusing width narrows down with an increase in the sheath flow rate, which validates that the experimental results are in good accordance with the numerical simulation results. Future work will be based on identifying how modifications to channel geometry and incoming sheath flow angle will affect flow focusing. We would also like to explore additional flow-focusing applications, such as mixing/separating particles and oil-in-water emulsion generation.

## Data Availability

All data provided in the present manuscript are available to whom it may concern.

## Supplementary Materials

The following supporting information can be downloaded at: https://www.mdpi.com/article/10.3390/mi14071388/s1. Figure S1 Full Width at Half Maximum Principle. Figure S2 Application of Full Width at Half Maximum Principle. Figure S3 Schematic illustration of symmetric hydrodynamic flow focusing; as shown in reference [50].
