# Supplementary material for: Processes for the 3D Printing of Hydrodynamic Flow-Focusing Devices
Source: Micromachines (Basel). 2023 Jul 7;14(7):1388. doi: 10.3390/mi14071388 (PMC10383660; doi:10.3390/mi14071388)
Supplement: Supplementary file 1 [file micromachines-14-01388-s001.zip › micromachines-2482772-supplementary.pdf]

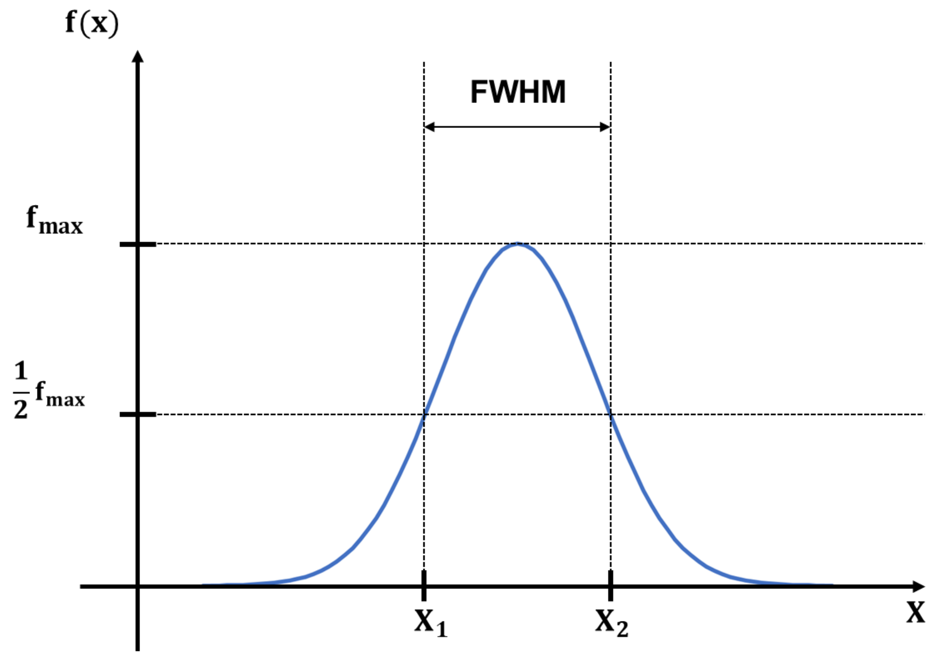

Figure S1 Full Width at Half Maximum Principle.

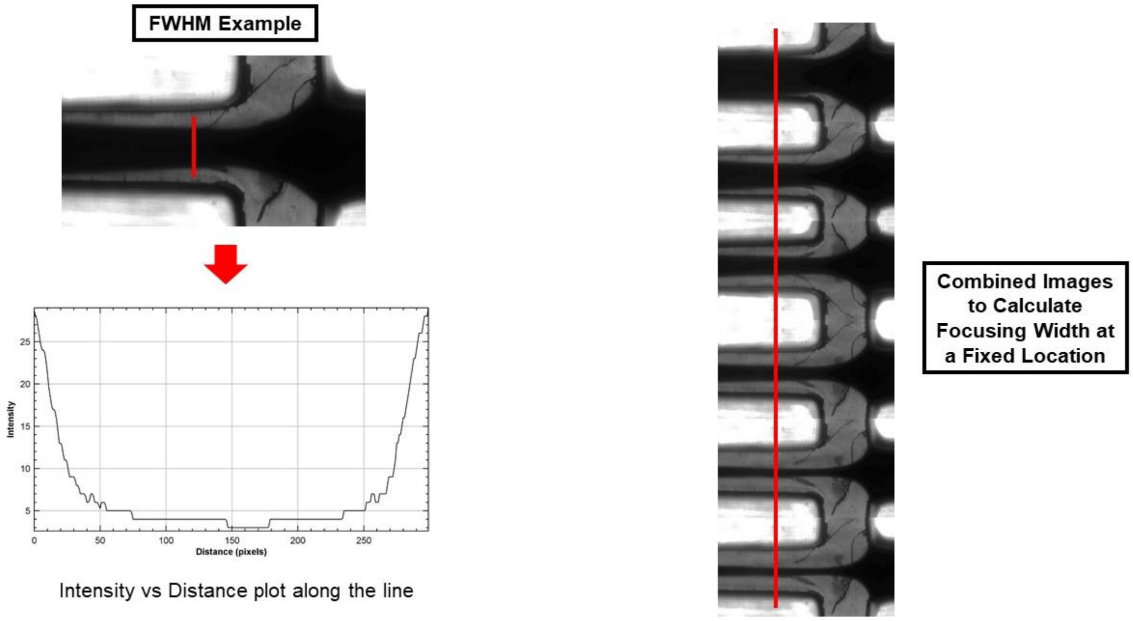

Figure S2 Application of Full Width at Half Maximum Principle.

Hydrodynamic Focusing Theory

The hydrodynamic flow focusing techniques provide control over the passage of samples through a microchannel of microfluidic device and has been successfully used in various applications, such as flow cytometry for cell/particle counting and sorting, diffusion-based mixers, micro flow switches, droplet generators and micro and nano-particle production.

This flow focusing technique depending on the applications can be classified as either: (i) multi-phase flow focusing (i.e. liquid–liquid or liquid–gas) or (ii) liquid single-phase flow focusing. It can be further classified into symmetric and asymmetric flow focusing for rectangular microchannels.

In two dimensional symmetric hydrodynamic focusing, the sample flow (supplied from the sample inlet) is squeezed by two sheath flows from side channels to align the sample flow across the centerline of the microchannel. In asymmetric hydrodynamic focusing, the sample flow is constrained by two unequal sheath flows (i.e. by sheath flows with different volumetric flow rates). The volumetric flow rates of the sample flow and sheath flows are generally controlled using multiple external pumps (e.g. syringe pumps) or pressure sources.

Microfluidic flows always occur at low Reynolds numbers and form a laminar and stable flow.
Therefore, the particles injected into them will follow well-defined streamlines. This stream of particles can be squeezed into a single file line by introducing another slower stream of sheath flow, this process is also known as hydrodynamic focusing.

Assuming consistent Reynolds number and length of the channel with minimal diffusion, the cross-sectional area of the focused stream can be predicted. However, the shape of the focused stream is difficult to predict as the forces controlling it involves complex calculations. In addition to the velocities at the inlets, the geometry of the microfluidic device has a major impact on the shape of the focused stream.

Effect of Velocity Ratio on Hydrodynamic Focusing:
A theoretical model for predicting the width of the two-dimensional hydrodynamically focused stream in a rectangular microchannel was developed. This analytical model presented in Wu et al. can be summarized as follows. In this study, the relationship between the width of the hydrodynamically focused stream and the volumetric flow rates of the inlet and side channels was determined. This relationship was further applied to symmetric ($Q_{s1} = Q_{s2} = Q_s$,) hydrodynamic focusing case.

This study made the following assumptions:

- The flow in the microchannels is steady, laminar and Newtonian.
- The fluid has the same density in the inlet channel, side channels, and outlet channel.
- The inlet, side, and outlet channels are of the same height.

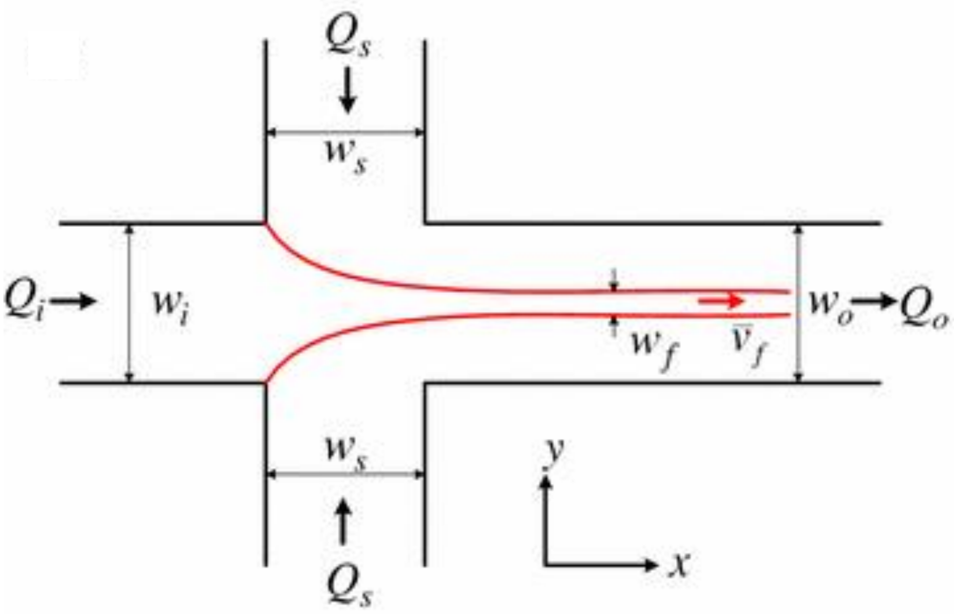

Figure S3 Schematic illustration of symmetric hydrodynamic flow focusing; as shown in reference [50].

According to the principle of mass conservation, the amount of fluid passing through the inlet channel must equal the amount of fluid passing through the dimension of the focused stream, i.e.

$$w_f = \frac{Q_i}{\bar{v}_f \times h} \tag{S1}$$

Moreover, the total amount of fluid passing through the outlet channel must equal the total amount of fluid supplied from the inlet and side channels, i.e.,

$$\bar{v}_f = \frac{Q_i + Q_{s1} + Q_{s2}}{w_o \times h} \tag{S2}$$

Therefore, the relationship between the width of the hydrodynamically focused stream (w_f) and the volumetric flow rates of the inlet channel (Q_i) and the side channels (Q_{s1} and Q_{s2}) can be expressed as

$$\frac{w_f}{w_o} = \frac{Q_i}{\gamma(Q_i + Q_{s1} + Q_{s2})} \tag{S3}$$

where the velocity ratio $\gamma = \bar{v}_f/\bar{v}_0$ is to be found, w_0 is the width of the outlet channel and $\bar{v}_f$ and $\bar{v}_o$ are the average flow velocities in the focused stream and the outlet channel, respectively.

Flow focusing in rectangular microchannel channels, the flow rates from the two side channels are equal, i.e. $Q_{s1} = Q_{s2} = Q_s$, as shown in Fig. 4. To predict the width of the symmetric
hydrodynamically focused stream, the unknown velocity ratio, γ , in equation (S3) must be found, as described in the paragraphs below.

The Reynolds number ($Re_{D_h} = \bar{v}_o D_h/\nu$, where D_h is the hydraulic diameter and ν is the kinematic viscosity) is generally very small in microfluidic devices. Since viscous effects dominate in low Reynolds number flows, the entrance length in the microchannel is very short. Therefore, the flow in the outlet channel can be assumed to be fully developed. Hence, the basic equation of the flow is given by

$$\frac{\partial^2 u}{\partial y^2} + \frac{\partial^2 u}{\partial z^2} = \frac{1}{\mu}\frac{\partial p}{\partial x} = const, \tag{S4}$$

where $u(y,z)$, $\partial p/\partial x$ and μ are the streamwise velocity, pressure gradient and fluid viscosity in the outlet channel, respectively. Imposing the no-slip condition on the channel wall, equation (S4) can be solved as

$$u(y,z) = \frac{4h^2}{\mu\pi^3}\left(-\frac{dp}{dx}\right)\sum_{n=0}^{\infty}(-1)^n \times \left\{1 - \frac{\cosh[(2n+1)\pi y/h]}{\cosh[(2n+1)\pi w_o/2h]}\right\}\frac{\cos[(2n+1)\pi z/h]}{(2n+1)^3} \tag{S5}$$

Equation (S5) is the well-known Poiseuille velocity profile for flow through a rectangular channel. In equation (S5), $-w_o/2 \le y \le w_o/2$ and $-h/2 \le z \le h/2$, where h is the height of the outlet channel. Integrating equation (S5) along the z-direction, the streamwise average velocity $\bar{u}(y)$ in the rectangular microchannel can be expressed as

$$\bar{u}(y) = \frac{1}{h}\int_{-h/2}^{h/2} u(y,z)dz = \frac{8h^2}{\mu\pi^4}\left(-\frac{dp}{dx}\right)\sum_{n=0}^{\infty}\frac{1}{(2n+1)^4} \times \left\{1 - \frac{\cosh[(2n+1)\pi y/h]}{\cosh[(2n+1)\pi w_o/2h]}\right\} \tag{S61}$$

The average velocities in the outlet channel, v_o, and the focused stream, $\bar{v}_f$, can be obtained from equation (S6). The velocity ratio, γ , is then given by

$$\gamma = \frac{\bar{v}_f}{v_o} = \frac{\frac{2}{w_f}\int_0^{w_f/2}\bar{u}(y)dy}{\frac{2}{w_o}\int_0^{w_o/2}\bar{u}(y)dy} = \frac{\{1-(192h/\pi^5 w_f)\}\sum_{n=0}^{\infty}\frac{1}{(2n+1)^5}\frac{\sinh[(2n+1)\pi w_f/2h]}{\cosh[(2n+1)\pi w_o/2h]}}{\{1-(192h/\pi^5 w_f)\}\sum_{n=0}^{\infty}\frac{\tanh[(2n+1)\pi w_o/2h]}{(2n+1)^5}} \qquad \text{(S7)}$$

Equation (S7) reveals that γ is dependent on the aspect ratio $\varepsilon(h/w_o)$ and the width of the focused stream, w_f. In case of pressure-driven flow, two cases exist: 1) $\varepsilon \rightarrow \infty$ where γ = 1.5 with $w_f/w_o \ll 1$ causing to form a parabolic velocity profile across channel width which will be independent of the position across channel height. 2) $\varepsilon \rightarrow 0$ where γ = 1.0 with $w_f/w_o \ll 1$ causing to form a parabolic velocity profile across channel height which will be independent of the position across channel width. However, the limitations of $w_f/w_o \ll 1$, $\varepsilon \rightarrow \infty$ and $\varepsilon \rightarrow 0$ could be avoided by using equations (S3) and (S7) to describe the hydrodynamic focusing effect in a rectangular channel.

<u>Millifluidic Device Fabrication - Process</u>

Before printing:

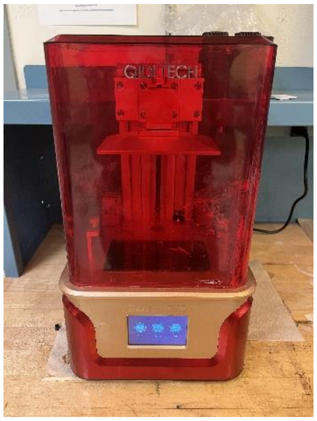

- Model the design in CAD software
- Make the base wide enough that there is plenty of room around each of the channel edges (usually 4-5mm is plenty)
- On the computer by the printer, import the model as an STL into Chitubox
- Reorient the model, then "slice" and save to USB drive
- Plug USB into 3D printer and select your design
- Place resin basin onto the printer
    - Fill with more resin if necessary
- Press "play" button on the printer screen
    - The display should show the estimated time remaining

After printing:

- Remove the printer build plate

  - Hold it over the resin basin to let excess resin drain back in
- Remove the print by first sliding a razer blade under the corner, and then gently use a putty knife
  - Sometimes it can be tricky to get the print off – may have to go slowly

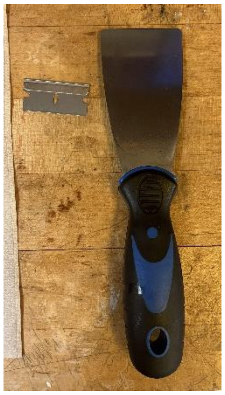

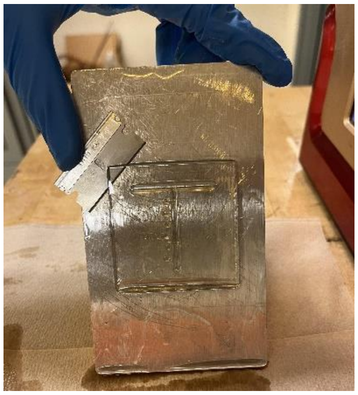

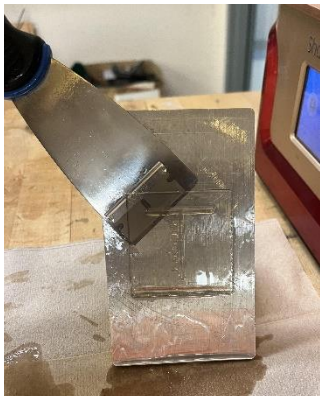

- Place print aside, and clean the build plate using Kimtech paper sheets
  - These are less abrasive than normal paper towels
- Gently wipe the remaining resin off of the print using the Kimtech sheets

Post-processing:

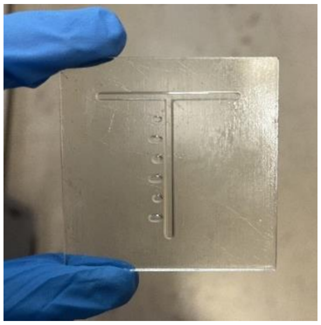

- Rinse the 3D print using isopropyl alcohol
  - Ethanol also works
  - Use a cotton swab and/or toothbrush to clean the channel edges
  - It's important to be thorough with the edges of the channels – any problems with the curing process usually occur there
- Place the print under the UV light for 1 hour
  - May have to do more time by trial and error – sometimes 1.5 or 2 hours helps the curing process later

- Place the print in the lab oven for 1 hour at Level 7
    - Usually 1 hour is enough, but may have to do more time by trial and error

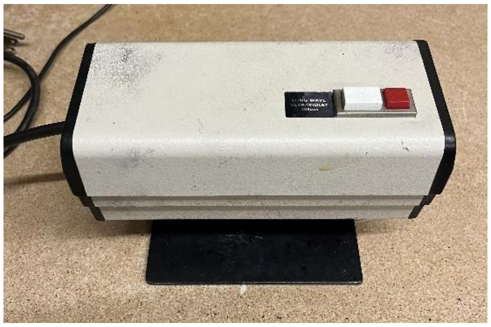

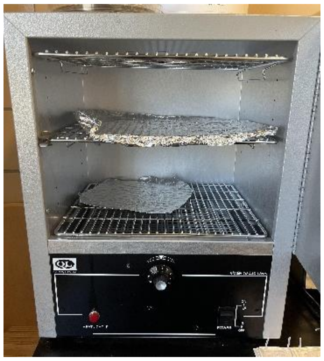

PDMS:

- In a small cup, mix parts A and B in a 10:1 ratio
- Place the 3D print in a petri dish and pour the PDMS over it
    - It can be helpful to push down on the 3D print with a stirrer in order to push as much of the air bubble as possible out from under the print
- Place the petri dish in the desiccator and turn on the vacuum pump to remove air
- Wait until the bubbles have fully moved to the surface
    - It sometimes helps to turn off the pump and open the valve periodically – the bubbles will pop when air gets let in
    - When the pump is on, the valve should have arrows pointing to the pump hose and the chamber, as seen below
- Put lid on petri dish and place in direct sunlight. Leave it there for at least 24 hours (longer is better)
    - If necessary, the petri dish can be placed in the oven at LOW heat to speed up the curing process

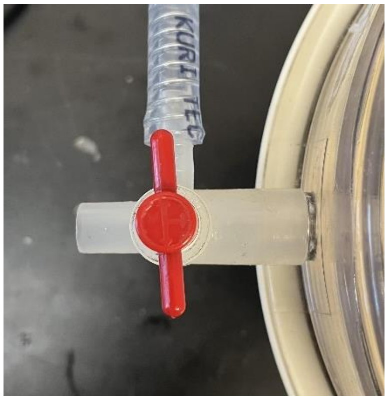

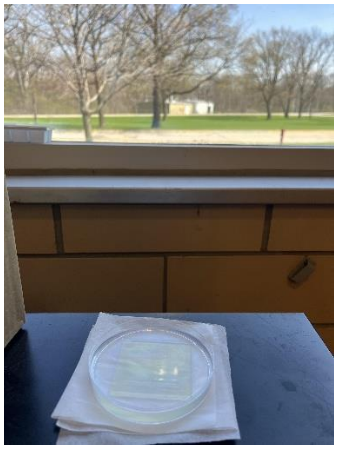

Bonding to glass:

- Prepare the glass slides by covering them in Scotch tape
  - This ensures that all dust is removed
- After the PDMS is fully cured, use an Exacto knife to cut out the PDMS in a rectangular shape, following the outline of the 3D print base
  - Generally, make the rectangular cut as large as possible to allow plenty of space around the channels for the PDMS to bond with the glass
- Carefully remove the PDMS by peeling back one corner, starting with tweezers
  - Wear gloves – it's important to keep the process as dust and dirt free as possible
- Place the PDMS on to a Kimtech sheet ("upside down," with the open channel facing up)
- Using a punch, remove PDMS at each of the channel edges where tubing will go
  - Lightly apply pressure and turn as you go to get a smooth cut
  - Remove the PDMS from within the punch between each cut using compressed air
- Carefully place the prepared PDMS, along with the taped glass slides, into a larger petri dish and take them to the room with the Plasma Cleaner

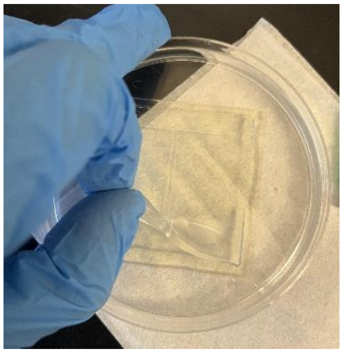
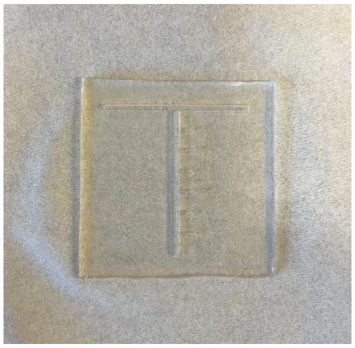
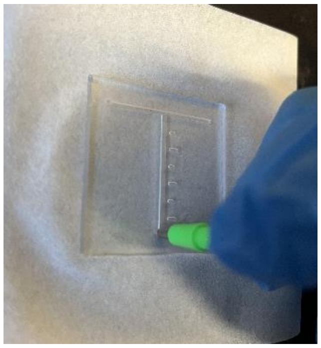

- Turn on the plasma oven and let sit for a minute or so
- Place the PDMS and glass slide on the glass piece and place into the chamber
  - The sides that are going to be bonded together should be facing up
- With the valve in the UP position, turn on the attached pump and let sit for a minute or so
- Open the oxygen tank, letting oxygen fill up the attached tube
  - The tube will fill up right away (you can tell when), so close the oxygen tank immediately
- Let the oxygen into the chamber by slowly turning the valve to the LEFT position (as shown)

- Return the valve to the UP position and let sit for a minute or so
- Turn the RF level knob to “HI,” for 40-45 seconds and then turn it off
    - o You should see the chamber turn to a purple-ish color. This is how you know it’s working

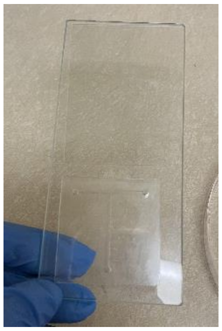

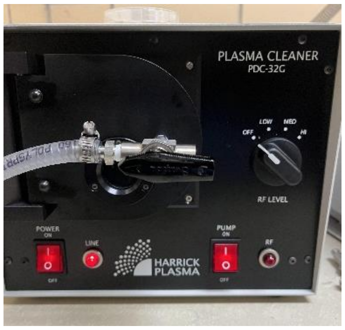

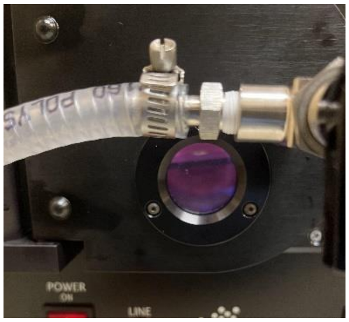

- Turn the RF level to OFF and turn off the attached pump
- Slowly turn the chamber valve to the RIGHT, letting the air out of the chamber
- Careful not to touch the upright side of the glass slide or the PDMS, place the surfaces together
    - o Once you do it, it cannot be repositioned
    - o You will be able to see the places the PDMS has bonded to the glass. Starting from the channel edges and moving outward, use tweezers or your finger to *gently* press the PDMS down on the glass
- Place device on a hot plate at 100C for 10 minutes to finish the bonding process

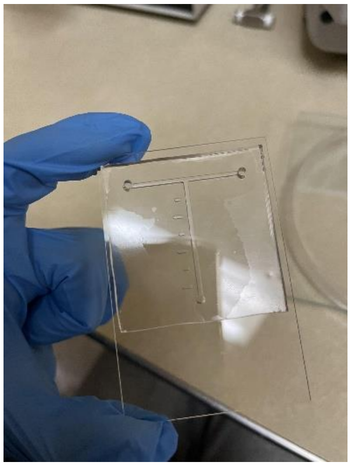

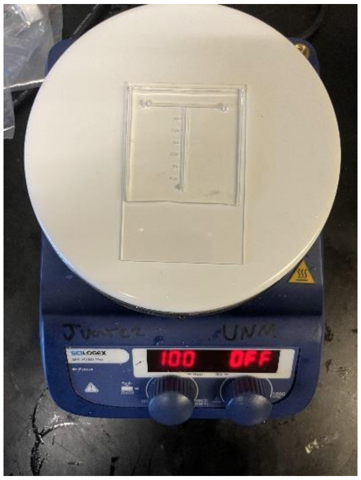

Tubing:

- Cut the tubing into small sections and use tweezers to place into the holes on the device
- In a small petri dish, mix a small amount of PDMS. Place into the desiccator to remove the bubbles
- Increase the viscosity of the PDMS by heating the petri dish on a hot plate
    - Only do this for 2-3 minutes (once the curing process has started, it will continue)
- Once the PDMS is thicker, use a cotton swab to place it around the tubing
    - Try to make it so it peaks up around the tubes as shown – this helps make sure the tubes don't leak
- Place PDMS over the channels as well
    - This makes it so the whole length of the channel looks the same when viewing under a microscope
- Place device on a hot plate and let sit until the PDMS is fully cured

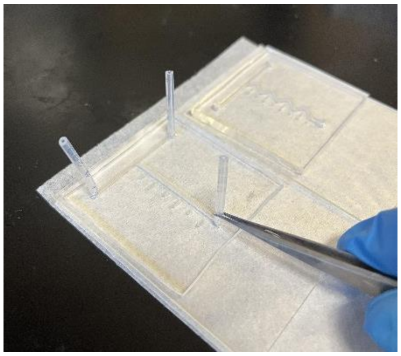

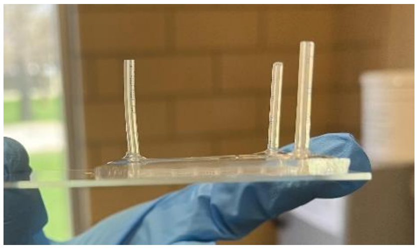

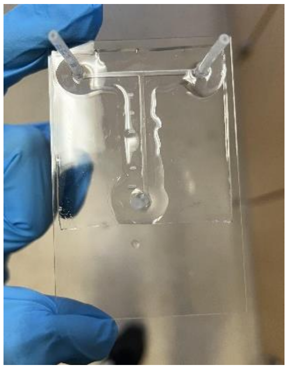

Tips:

- After cutting out the 3D print, mix and pour new PDMS into the petri dish – the mold can be reused to make new devices
- If the PDMS isn't curing, you may need to increase more time for the UV light exposure or time in the lab oven (post-processing is important!)
- If the process isn't working, it can be helpful to just go back and restart the process in order to ensure that every step is done correctly and thoroughly

Reference

50. Lee, G.B.; Chang, C.C.; Huang, S.B.; Yang, R.J. The hydrodynamic focusing effect inside rectangular microchannels. *J. Micromechanics Microengineering* **2006**, *16*, 1024.
